# miR-449a targets Flot2 and inhibits gastric cancer invasion by inhibiting TGF-β-mediated EMT

**DOI:** 10.1186/s13000-015-0435-5

**Published:** 2015-11-14

**Authors:** Qian Li, Jie Peng, Xinhua Li, Aimin Leng, Ting Liu

**Affiliations:** Department of Gastroenterology, Xiangya Hospital, Central South University, Changsha, Hunan 410008 P. R. China

**Keywords:** miR-449a, FLOT2, Gastric cancer, TGF-β-mediated-EMT

## Abstract

**Background:**

Flot2, a highly conserved protein of the SPFH domain containing proteins family, has recently been identified as oncogene to be involved in the tumorigenesis and metastasis of several cancers including gastric cancer. However, the underlying molecular mechanism of Flot2 in gastric cancer (GC) is largely unknown.

**Methods:**

qRT-PCR and western blot was performed to detect miR-449a and Flot2 expression in GC cell lines and Normal human gastric epithelial cells. Then, luciferase reporter assay was used to elucidate whether Flot2 is a target gene of miR-449a. Finally, the roles and mechanism of miR-449a in regulation of tumor invasion were further investigated.

**Results:**

In this study, miR-449a expression was downregulated and Flot2 was upregulated in all GC cell lines as compared with that in GES-1. luciferase reporter assay identified Flot2 as a novel direct target of miR-449a. miR-449a regulated GC cell invasion by suppressing Flot2 expression. Expression analysis of a set of epithelial-mesenchymal transition (EMT) markers showed that miR-449a reduced the expression of mesenchymal markers (vimentin and N-cadherin) and induced the expression of epithelial marker (E-cadherin), which was consistent with silenced Flot2. Moreover, Flot2 is necessary for TGF-β-induced EMT in GC cells.

**Conclusions:**

Our results demonstrated that miR-449a suppressed Flot2 expression results in decreased cell invasion through repressing TGF-β-mediated-EMT, and provides a new theoretical basis to further investigate miR-449a-regulated Flot2 as a potential biomarker and a promising approach for GC treatment.

## Background

Gastric cancer (GC) is the second leading cause of cancer-related deaths worldwide with 934,000 new cases occurring each year [1]. Despite improvements in the diagnosis and treatment of GC, the overall 5-year survival rate of patients with GC and even resectable disease has a 50–90 % risk of recurrence and death [2]. Therefore, it is essential to elucidate the molecular mechanisms of GC proliferation and metastasis, which will provide important insights and help us find new diagnostic and therapeutic approaches to this disease and improve the prognosis of GC patients.

Lipid rafts function as physical platforms for various molecules that are involved in a variety of biologic processes by tethering growth signaling molecules linked to signal transduction pathway and has been reported to be involved in initiation and progression of human cancers [3–6]. Flotillin-2 (Flot2), a member from flotillin family, is a marker of lipid rafts and reported to play key roles in the development and progression of human malignant tumors [7]. Wang et al. recently showed that high Flot2 expression in human non-small cell lung cancer and its correlation with tumor tumorigenesis and patient survival [8]. Rickman et al. revealed that Flot2 has a predictive value for the development of metastases in head and neck cancer [9]. Up-regulation of Flot2 is associated with disease progression and poor clinical survival in renal cell carcinoma [10], cervical carcinoma [11] and breast cancer [12]. Recently, siRNA-mediated Flot2 downregulation was reported to inhibit cell proliferation and invasion in gastric carcinoma [13], however, the underlying molecular mechanism of Flot2 in cell invasion of GC is largely unknown.

MicroRNAs (miRNAs), as a class of small (22-nucleotide) non-coding RNAs, have been identified to be aberrantly expressed in several human malignancies [14]. miRNAs regulate gene expression by binding to the 3′untranslated region (3′-UTR) of their target mRNAs, modulating mRNA stability and/or translation [15]. Previous studies have identified a number of miRNAs that show aberrant expression in GC. Xie et al. revealed 14 upregulated miRNAs (including miR-21, miR-26b and miR-30b) and 19 downregulated miRNAs (including let-7i, miR-7 and miR-622) which contributes to gastric cancer development and progression by using miRNA microarray profiling [16]. The expression of miR-141 was reported to be significantly reduced in GC and was significantly correlated with a more aggressive phenotype of GC in patients [17]. miR-153, as an independent prognostic marker for predicting survival of gastric cancer patients, is downregulated in GC and promote gastric cancer cell migration and invasion, by inhibiting SNAI1-induced EMT [18]. miR-449a was downregulated in gastric cancer cell lines and gastric cancer tissues [19, 20], and inhibits proliferation and induces apoptosis by directly repressing E2F3 [19]. However, the effects and mechanism of miR-449a on GC cell invasion remains unclear.

In this study, luciferase reporter assay identified Flot2 as a novel direct target of miR-449a. miR-449a mediated Flot2 suppression resulted in reduced GC cell invasion via repressing TGF-β-induced EMT.

## Methods

### Cell culture and transfection

Normal human gastric epithelial cells GES-1 were obtained from the Cell Bank of Xiangya Central Laboratory, Central South University (Changsha, Hunan, China). The gastric cancer cell line NCI-N87 (well differentiated), MGC-803 (poorly differentiated) and human gastric adenocarcinoma cell lines SGC-7901 (moderately differentiated) were obtained from the Cell Bank of the Chinese Academy of Sciences (Shanghai, China). The cells were maintained in DMEM medium, supplemented with 10 % fetal bovine serum (FBS; Hyclone, USA), 100 U/mL penicillin, and 100 μg/mL streptomycin. The cells were incubated in an atmosphere of 5 % CO_2_ at 37 °C.

miR-449a mimics, negative controls (NC), and siRNA targeting human Flot2 mRNA were designed and synthesized by Shanghai GenePharma Company (Shanghai, China). Full length Flot2 cDNA (GenBank accession number NM_004475.2) was cloned into the pcDNA3.1 vector (Addgene, Cambridge, MA, USA). The constructed plasmid was verified by DNA sequencing. In miR-449a mimics group, MGC-803 cells were transfected with miR-449a mimic or miR-Ctrl (50 nM; GenePharma, Suzhou, China) using Lipofectamine 2000 reagent (Invitrogen). In siRNA group, MGC-803 cells were transfected with the Flot2 siRNA (100 ng; FulenGen, Guangzhou, China) using Lipofectamine 2000 reagent (Invitrogen). The cells were harvested for assays 48 h after transfection.

### qRT-PCR

The PCR amplification for the quantification of the miR-449a and U6 was performed using TaqMan miRNA Reverse Transcription Kit (Applied Biosystems, Foster City, CA, USA) and TaqMan Human MiRNA Assay Kit (Applied Biosystems, Foster City, CA, USA). The relative expression of miR-449a was shown as fold difference relative to U6. The PCR amplification for the quantification of the Flot2 and GAPDH mRNAs was performed using an ABI PRISM 7300 Sequence Detection System (Applied Biosystems, Foster City, CA, USA) and a SYBR®Premix Ex Taq™ ii (Perfect Real Time) Kit (Takara Bio, Shiga, Japan) as described previously [10].

### Western blot

Whole cell extracts were prepared with a cell lysis reagent (Sigma-Aldrich, St. Louis, MO, USA) according to the manual, and then, the protein was quantified by a BCA assay (Pierce, Rockford, IL, USA). Then, the protein samples were separated by SDS-PAGE (10 %) and detected by Western blot using polyclonal (rabbit) anti-Flot2, anti-E-cadherin, anti-N-cadherin and anti-Vimentin antibody (Santa Cruz Bio-technology, Santa Cruz, CA, USA). Goat anti-rabbit IgG (Pierce, Rockford, IL, USA) secondary antibody conjugated to horseradish peroxidase and ECL detection systems (SuperSignal West Femto, Pierce) were used for detection.

### Luciferase reporter assay

The 3′-UTR sequence of Flot2 was amplified from normal human genomic DNA and subcloned into the pmirGLO luciferase reporter vector (Promega). MGC-803 cells (3.5 × 10^4^) were seeded in triplicate in 24-well plates and cotransfected with wild-type (WT) or mutant (Mut) 3′-UTR vectors and miR-449a mimics using Lipofectamine 2000. After 48 h, the cells were assayed for luciferase activity using the Dual-Luciferase Reporter Assay System (Promega) by following the manufacturer’s instructions. The firefly luciferase activities were normalized to Renilla luciferase activity. The firefly luciferase activity of the cells that were transfected with miRNA mimics or inhibitors is represented as the percentage of activity relative to that of cells that were transfected with negative controls. All experiments were performed in triplicate.

### Cell invasion assay

The capability of cell invasion was examined by transwell invasion assay. Cells were cultivated to 80 % confluence on the 12-well plates. Then, we observed the procedures of cellular growth at 72 h. All the experiments were repeated in triplicate. The transwell migration chambers were used to evaluate cell invasion. Then cells invasing cells across the membrane were counted under a light microscope.

### Statistical analysis

Each experiment was repeated at least three times. Data were shown as mean ± s.d and analyzed using SPSS 18.0. Statistical comparisons between groups were analyzed using Student’s *t*-test and a two-tailed *p* < 0.05 was considered to indicate statistical significance.

## Results

### The expression of miR-449a and Flot2 in GC cell lines

**Fig. 1:**
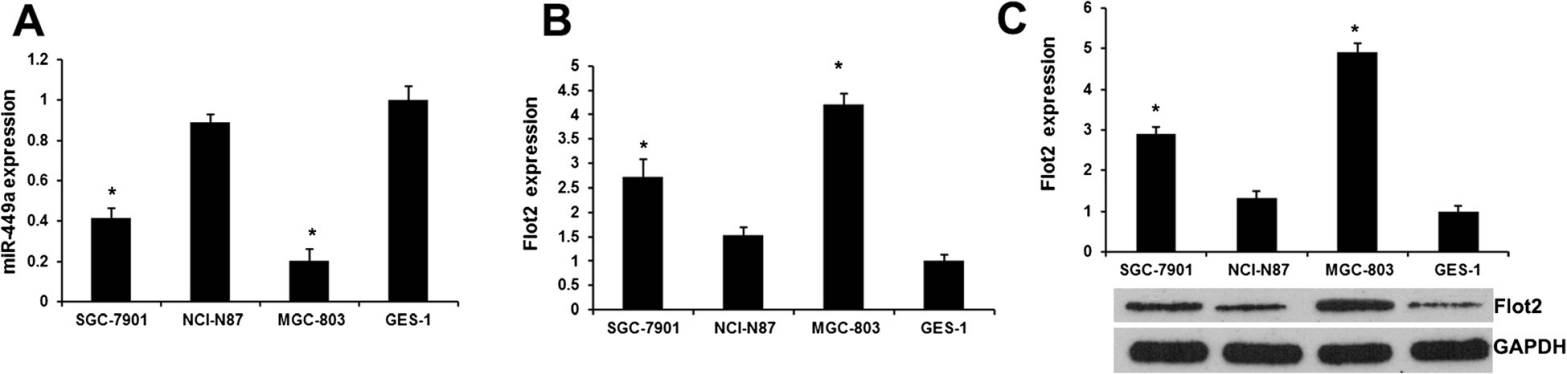
The expression of miR-449a and Flot2 in NPC cell lines. **a** qRT-PCR analysis revealed the miR-449a expression in normal human gastric epithelial cells GES-1 and gastric cancer cell lines (SGC-7901, NCI-N87 and MGC-803). **b** qRT-PCR analysis revealed the Flot2 expression in normal human gastric epithelial cells GES-1 and gastric cancer cell lines (SGC-7901, NCI-N87 and MGC-803). **c** Western blot analysis revealed the Flot2 expression in normal human gastric epithelial cells GES-1 and gastric cancer cell lines (SGC-7901, NCI-N87 and MGC-803). Each bar represents the mean of three independent experiments. * *P* < 0.01 versus GES-1 cell line

We first employed qRT-PCR to detect miR-449a levels in normal human gastric epithelial cells GES-1 and gastric cancer cell lines (SGC-7901, NCI-N87 and MGC-803). Consistent with previous study [19], the miR-449a expression was downregulated in all GC cell lines as compared with that in GES-1 (Fig. 1a), indicating that miR-449a may function as a tumor suppressor in GC cells.

We next assayed the Flot2 expression levels in normal human gastric epithelial cells GES-1 and gastric cancer cell lines (SGC-7901, NCI-N87 and MGC-803) using qRT-PCR and western blot assay. Figure 1b and c showed that the Flot2 expression was upregulated in all GC cell lines as compared with that in GES-1, indicating that Flot2 was an oncogene in GC cells.

### miR-449a directly targeted Flot2

**Fig. 2:**
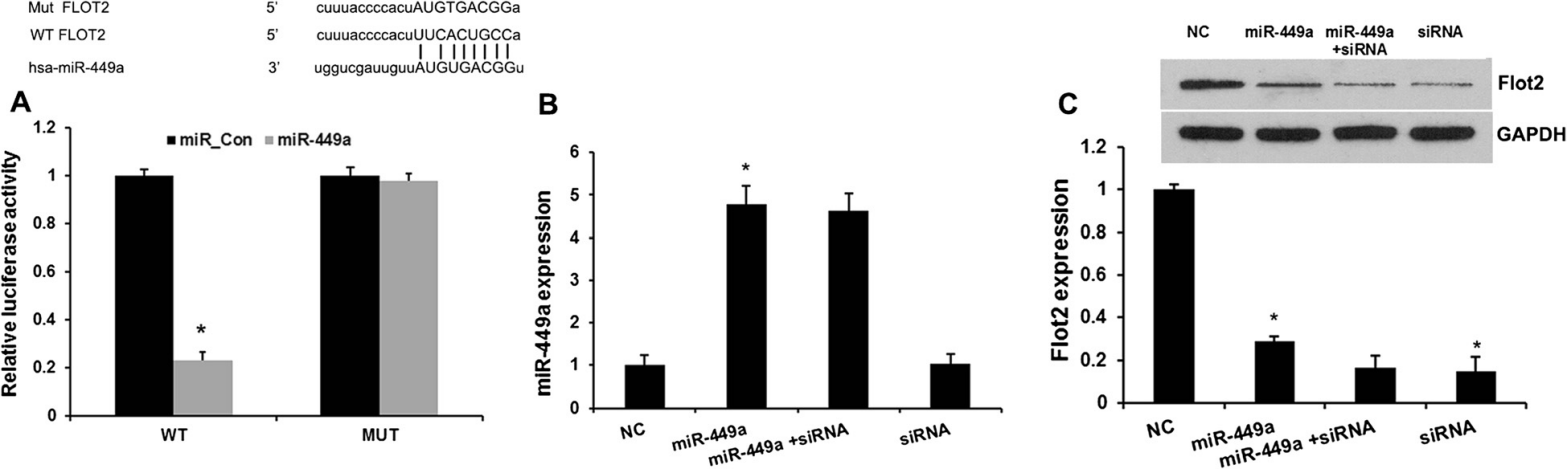
miR-449a directly targeted Flot2. **a** Representative diagram of the predicted wild-type (WT) or mutant (Mut) binding site of miR-449a in the 3'- untranslated region (UTR) of Flot2 mRNA. The luciferase reporter plasmid containing the WT or Mut Flot2 3′-UTR was cotransfected into HEK293T cells with miR-449a mimics. Luciferase activity of the cells was assayed at 48 h after transfection, and the values were normalized to the normal control values. *P <0.01 (compared with the control). **b** qRT-PCR analysis revealed the effects of Flot2 siRNA and miR-449a mimics on the expression level of miR-449a. **c** Western blot analysis revealed the effects of Flot2 siRNA-810 and miR-449a mimics on the expression level of Flot2. Error bars represent ± S.E. and *, *p* < 0.01 versus control and NC

To elucidate whether Flot2 is a potential downstream target gene of miR-449a in GC cells, we constructed luciferase reporter vectors containing the wild-type (Wt) or mutant (Mut) miR-449a target sequences of the Flot2 3′-UTR (Fig. 2a). Overexpression of miR-449a significantly inhibited the luciferase activity of the Wt Flot2 3′-UTR reporter gene but not the Mut reporter gene (Fig. 2a).

Next, we transfected Flot2 siRNAs in MGC-803 cells. Western blot analysis revealed Flot2 expression were significantly downregulated by Flot2 siRNA (*P* < 0.05). In addition, overexpression of miR-449a markedly reduced the expression of Flot2 (Fig. 2c), but silenced Flot2 did not affect miR-449a expression (Fig. 2b). These results demonstrated that Flot2 is a direct target of miR-449a in GC cells.

### Flot2 involves in miR-449a-regulated GC cell invasion

**Fig. 3:**
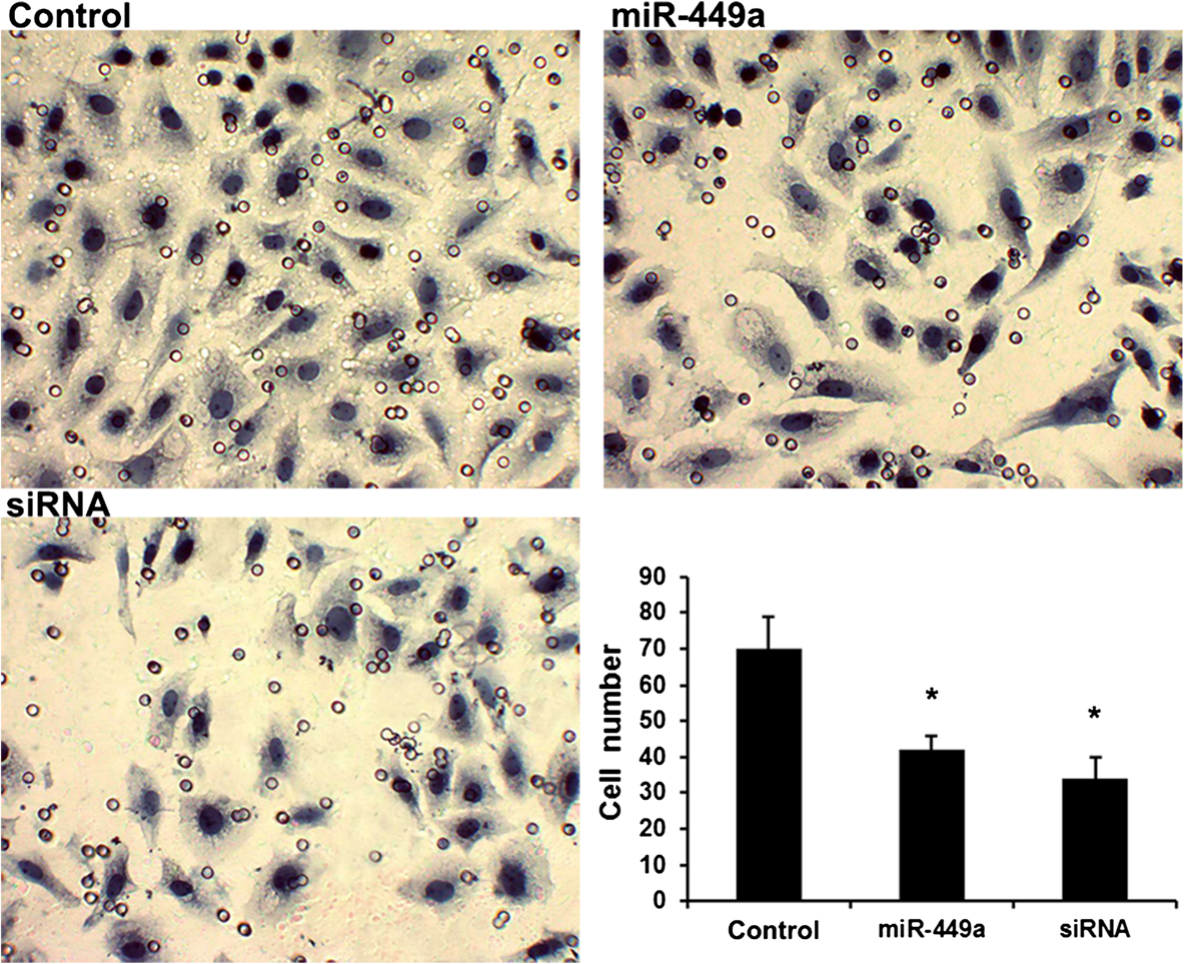
Flot2 involves in miR-449a-regulated BC cell invasion. The transwell invasion assay revealed the effects of Flot2 and miR-449a on BC cell invasion. Error bars represent ± S.E. and *, *p* < 0.01 versus control

To determine the role of Flot2 and miR-449a in the GC cell invasion, MGC-803 cells were transiently transfected with Flot2 siRNA (siRNA group) or miR-449a mimic (miR-449a group). Consistent with the effects induced by miR-449a mimics, knockdown of Flot2 significantly suppressed the cell invasion (Fig. 3), indicating that miR-449a suppress GC cell invasion by repressing Flot2 expression.

### MiR-449a reduces EMT of GC cells by suppressing Flot2 expression

**Fig. 4:**
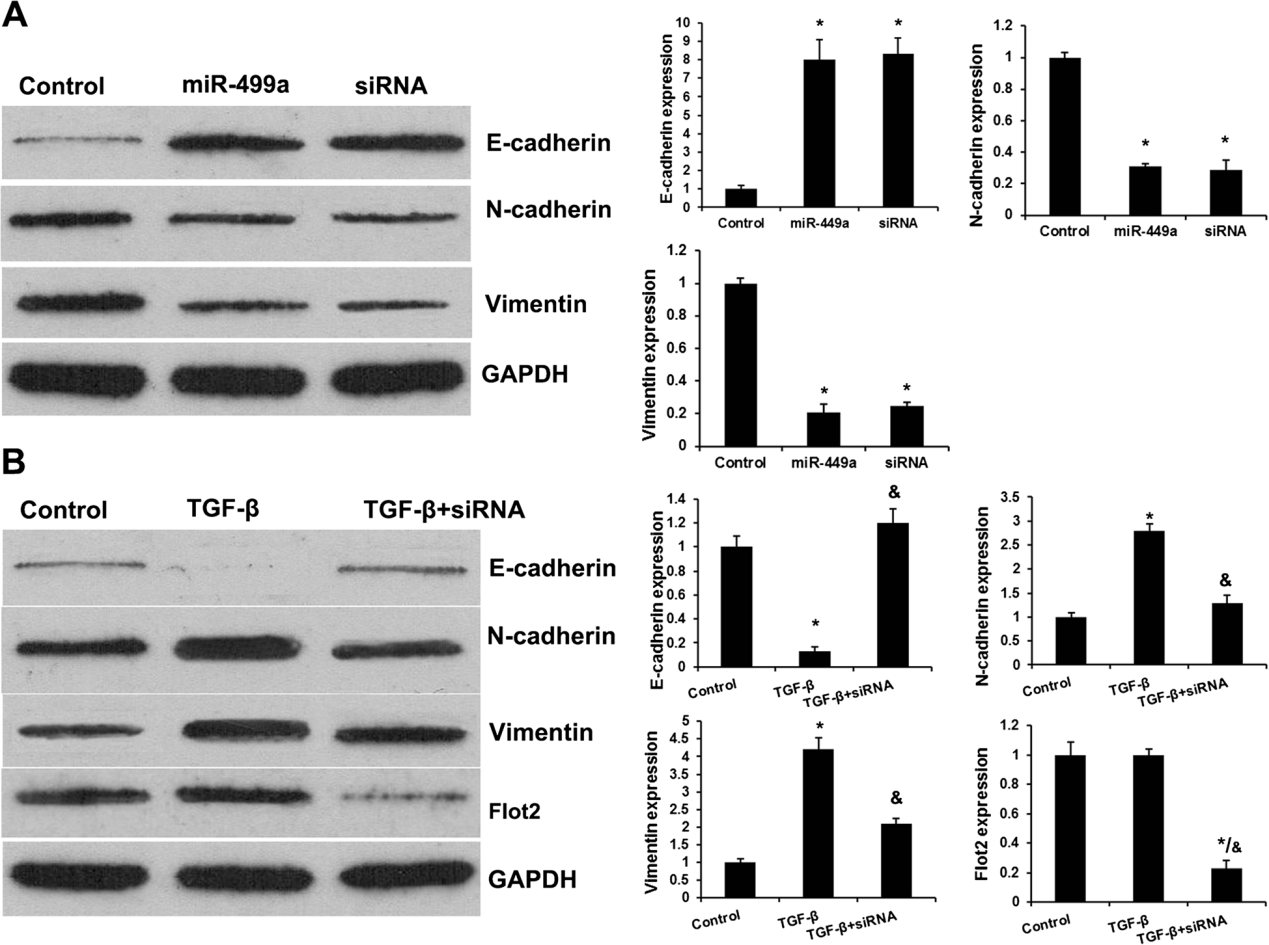
MiR-449a repressed Flot2 regulates TGF-β-mediated EMT in GC. **a** MiR-449a induces EMT of GC cells by suppressing Flot2 expression. Western bolt analysis revealed the effects of miR-449a and Flot2 on EMT-relative protein expression. **b** Upregulation of Flot2 is necessary for TGF-β-induced EMT in GC. Western bolt analysis revealed the effects of TGF-β and Flot2 siRNA on EMT-relative protein expression. Error bars represent ± S.E. and *, *p* < 0.01 versus control. &, *p* < 0.01 versus TGF-β group

EMT has been identified as a key role in the invasion of various cancer cells by the transformation of polarized and adherent epithelial cells into motile and invasive mesenchymal cells. Here, to explore protein regulated by miR-449a in the EMT process, we investigated the expression of three EMT related proteins, E-cadherin, N-cadherin and Vimentin by Western blot. MGC-803 cells were transfected with NC, miR-449a mimics and Flot2 siRNA. Results indicated the expression of E-cadherin was increased in miR-449a mimics group compared with control groups (Fig. 4a). Moreover, E-cadherin expression in siRNA group was higher than that in control and similar with that in miR-449a group. N-cadherin and Vimentin was downregulated significantly in miR-449a group. Moreover, N-cadherin and Vimentin expression in Flot2 siRNA group were higher than that in control group and similar with that in miR-449a group. This indicated miR-449a-repressed Flot2 expression reduced epithelial–mesenchymal transition (EMT) with elevated expression of E-cadherin and reduced expression of Vimentin and N-cadherin in MGC-803 cells.

### Upregulation of Flot2 is necessary for TGF-β-induced EMT in GC

TGF-β is the most potent inducer of EMT in epithelial cancers [21]. To assess the effect of TGF-β on EMT of gastric cancer cells, MGC-803 cells were treated with TGF-β (20 ng/mL) and harvested at 24 h. We found that TGF-β led to significant induction of mesenchymal markers Vimentin and N-cadherin. Meanwhile, expression of E-cadherin, an epithelial marker, was decreased after TGF-β treatment as shown by western blotting analyses (Fig. 4b). It was recently reported that Flot2 is an indispensable member for TGF-β signaling in nasopharyngeal carcinoma [22]. So we hypothesized that Flot2 might be associated with TGF-β signaling in EMT. Next, Flot2 siRNA was added into the TGF-β cultured GC cells, and the expression of a set of epithelial-mesenchymal transition (EMT) markers were analyzed by western blot. Figure 4b showed that Flot2 siRNA partially rescued the TGF-β-reduced expression of epithelial marker (E-cadherin) and TGF-β-induced expression of mesenchymal markers (Vimentin and N-cadherin) in TGF-β-siRNA group. Then we are curious whether TGF-β stimulation increased Flot2 expression. To our surprise, no significant changes were found on the Flot2 expressions under TGF-β stimulation.

Taken together, these experiments demonstrate that upregulation of Flot2 is necessary for TGF-β-induced EMT in MGC-803 cells.

## Discussion

Accumulated studies have shown that altered miRNA expression is closely associated with gastric cancer progression and poor prognosis [23–25], and they are involved in the cancer development and progression through binding at the 3′ UTR of cancer related genes’ mRNAs and thereby controlling their expression. For example, miR-133a is downregulated in GC and functions as a tumor suppressor in vitro and in vivo partly by repressing IGF1R [26]. microRNA-29c mediates initiation of gastric carcinogenesis by directly targeting ITGB1 [27]. MicroRNA-506 inhibits gastric cancer proliferation and invasion by directly targeting Yap1 [28]. Here, we demonstrated the mechanism of miR-449a and its novel specific target Flot2 on GC invasiveness.

Deregulated FLOT2 is associated with progression and poor survival in numerous types of cancer [10, 12, 22, 29], making it a critical regulator of tumor initiation and prognosis. Previous studies have suggested that the upregulation of Flot2 protein is significantly correlated with cancer progression and poor prognosis in gastric carcinomas [13], by regulating gastric cancer cell metastasis . However, the underlying mechanism by which this occurs is still unclear. In this study, we found that Overexpression of miR-449a significantly inhibited the luciferase activity of the Wt Flot2 3′-UTR reporter gene but not the Mut reporter gene and overexpression of miR-449a markedly reduced the expression of Flot2, but silenced Flot2 did not affect miR-449a expression, indicating that Flot2 is a direct target of miR-449a in GC cells.mediated suppressed Flot2 expression in GC. Moreover, transwell invasion assay showed that miR-449a inhibited GC cell invasion by suppressing Flot2 expression.

The epithelial-mesenchymal transition (EMT) has been associated with the acquisition of migration, invasiveness, and metastasis traits. The preponderance of evidence indicates that inappropriate activation of EMT in cancer results in driving cancer cell migration, invasion, and ultimately metastasis [21]. During EMT, the epithelial protein level, such as E-cadherin, are downregulated, while mesenchymal protein such as N-cadherin and vimentin are upregulated [30].

To determine the role and potential mechanism of Flot2 in cell invasiveness, we analyzed the effects of overexpressed miR-449a and silenced Flot2 on the expression of epithelial marker (E-cadherin) and induced expression of mesenchymal markers (Vimentin and N-cadherin) in GC cells. Our data indicate that overexpressed miR-449a and loss of Flot2 in GC cells results in an more epithelial morphology with upregulation of E-cadherin and downregulation of vimentin and N-cadherin.

During tumor progression, EMT can be induced by transforming growth factor-β (TGF-β) signal that epithelial cells receive from their microenvironment [31]. Previous studies showed that Flot2 is an indispensable member for TGF-β signaling in nasopharyngeal carcinoma [22]. Therefore, we hypothesized that the upregulated Flot2 might be critical in invasion of GC associated with TGF-β mediated EMT. In this study, silenced Flot2 reduced the degree of EMT transformation induced by TGF-β. However, surprisingly there are no significant changes were found on Flot2 expressions under TGF-β stimulation. Thus we speculate Flot2 is regulated independent of TGF-β but necessary for TGF-β dervided EMT in GC cells.

## Conclusions

In summary, we demonstrated the important role of miR-449a mediated Flot2 suppression that subsequently disarranged TGF-β induced epithelial mesenchymal transition within gastric cancers, thus providing a potential therapeutic target in gastric cancer therapy.
